# Calibration – an under-appreciated component in the analytical process of the medical laboratories

**DOI:** 10.1515/almed-2023-0127

**Published:** 2023-11-13

**Authors:** Oswald Sonntag, Tze Ping Loh

**Affiliations:** Independent Consultant, Eichenau, Germany; Clinical Chemistry Division, Department of Laboratory Medicine, National University Hospital, Singapore, Singapore

**Keywords:** calibration, linearity, patient safety, quality control

## Abstract

Calibration of an analytical measurement procedure is an important basis for the reliability of patient results. Many publications and as well as procedures on how to estimate quality control and interpret those results have been become available over the years. In this publication we are focusing on the critical part of the calibration as there are no clear communication or guidelines on how to perform it. Usually only the recommendation of the reagent or instrument manufacturer is available. We would like to point out this gap to invite for a discussion and improvement of the current situation.

## Introduction

Calibration is the cornerstone of any quantitative measurement procedure and an integral part of the daily routines in all laboratories. It describes the relationship between the signal intensity and the concentration of a measurand in the measurement procedure through the use of a material (calibrator) with defined concentration [1], [2], [3]. Following the construction of the calibration curve, the concentration of an unknown sample can be estimated by subjecting it to the measurement procedure and applying the measured signal on the calibration curve to interpolate its concentration.

With appropriate traceability chain, calibration provides a link between the measurement results of a patient and a higher order reference material or measurement procedure. Ideally, a primary reference material and a primary reference measurement procedure are used to anchor the apex of the traceability chain, which is important for standardization of measurement procedures. Nonetheless, such traceability chain has not achieved commonplace in clinical laboratory practice, although there are dedicated activities towards this goal.

Despite the clear importance of calibration on the production of reliable laboratory results, the details of proper calibration procedures are seldom discussed in clinical laboratory guidelines. This has left the instructions of the manufacturer of the measurement procedure as the primary source of information. Our purpose is to discuss aspects of the calibration process that may be less well described in the literature.

## An example from a commercial measurement procedure

The following information is a typical ‘instructions for use’ provided by commercial manufacturers of routine (automated) clinical biochemistry measurement procedures.

Calibrator 1: distilled water=reagent blank.

Calibrator 2: calibrator which is given by the manufacturer.

Calibration type: linear.

Calibration frequency: 2-point calibration after reagent lot change and when quality control procedures require this.

Traceability: the method was standardized against reference method Z and the primary reference material Y was used.

Routinely, only a single calibrator is used, and only a single measurement is made for the actual calibration procedure, despite the fact that measurement of calibrators is also associated with measurement uncertainties. The impact of variation associated with calibration is demonstrated through the following series of figures.

**Figure 1:**
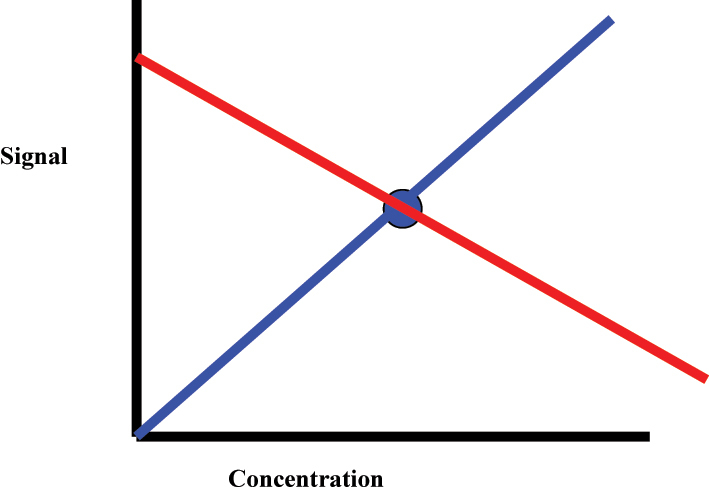
A single-point calibration where a regression can be constructed in any direction through the single calibration measurement. The red and blue represent two (out of an infinite) possible negative and positive calibration regression.

A linear regression requires a minimum of two points to be constructed, i.e. two points is needed to connect a straight line. When a single calibrator is used, any regression curve can be constructed through the single calibrator measurement (Figure 1) since there is a lack of another point to anchor the direction. Consequently, no predictable relationship can be inferred between the signal and concentration.

**Figure 2:**
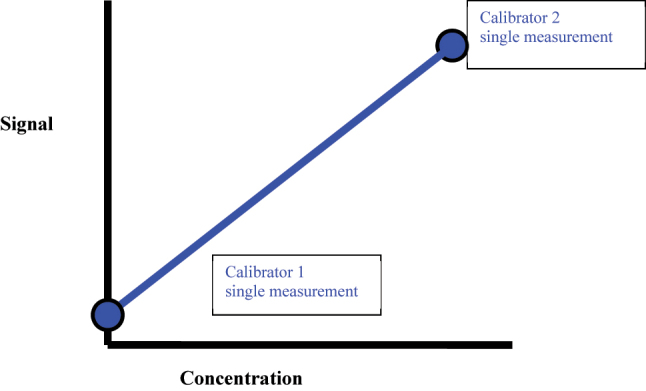
A linear regression can be constructed with two-point calibration that describes the direction and slope between the signal and calibrator concentration.

When two calibration measurements are made, a linear regression can be constructed that describes the direction and magnitude (slope) of the relationship between the signal and concentration (Figure 2). More complex relationship between signal and calibrator concentration requires more measurements (calibration points) to describe. For example, the minimum number of calibration points for a linear curve is two, for a exponential curve is three and for a sigmoidal curve is four [4]. A larger number of calibration points will better characterize the relationship between the signal and the concentration. It is necessary to employ a larger number of calibrators during method development to ensure the calibration curves are properly characterized before reducing the number of calibrators, providing it does not compromise the reliability of the measurements [5].

**Figure 3:**
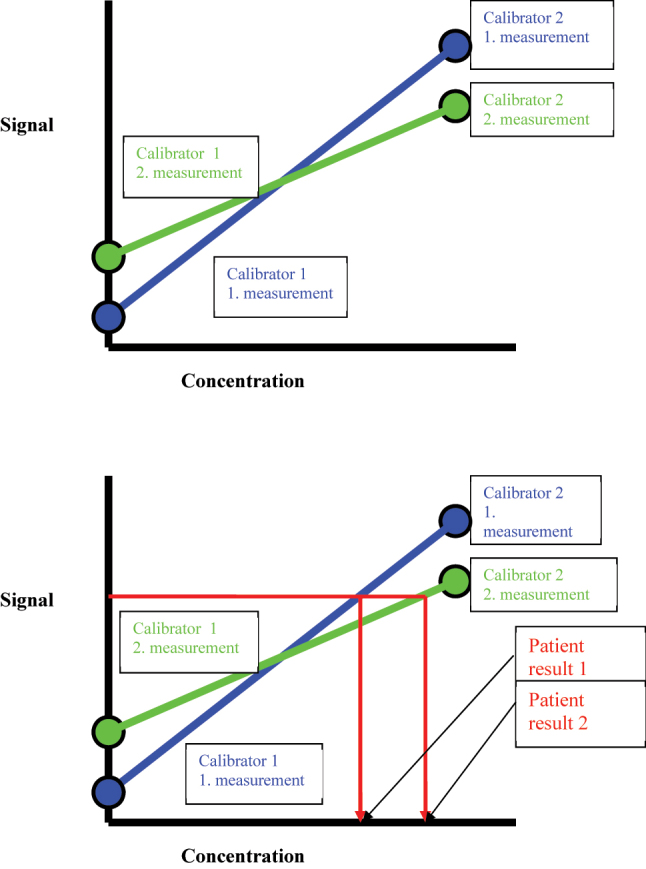
Two calibration curves constructed from two separate single measurements of the calibrators with different slope and intercept that will manifest as a difference in observed measurement when subjected to the same patient sample.

Besides the number of calibration point, another important factor affecting the reliability of a calibration curve is the variation (uncertainty) associated with its measurement. The measurement of a calibration material (calibrator) is associated with the same analytical variation (and hence measurement uncertainty) as other samples of the same matrix. A single measurement is associated with a larger uncertainty compared to the average of a number of replicate measurements. When the data from a single measurement is used to construct the calibration curve, it will be associated with a larger between-calibration variation. This may manifest as an analytical shift in the observed measurement (Figure 3).

In routine clinical laboratory, the reliability of the calibration curve is monitored by the measurement of internal quality control. This is practiced the assumption that in the absence of clinically significant matrix effects, an in-control quality control measurement is indicative that a between calibration variation is within acceptable limits for patient result reporting.

For most routine clinical biochemistry measurement procedures, only one calibration point is carried out in the laboratory under routine conditions to determine the measurement signal for the reagent blank (calibrator 1) and the measurement signal for the manufacturer calibrator (calibrator 2). Replicate measurements of the calibrators (reagent blank and manufacturer calibrator) are usually not made due to cost reasons. The subsequent quality control measurements are relied upon to reveal any gross errors in the calibration.

However, this may not always hold true because the quality control materials supplied by the reagent manufacturer can obscure the calibration error. This is particularly true when the source material for the quality control and the calibrator are mostly identical and are adjusted to the reagent. There is an increased risk of accepting an erroneous calibration curve and the consequent increased risk of erroneous patient results. This risk may be mitigated by using third-party quality control materials as recommended by the ISO 15289: 2022 – 7.3.7.2. [6]: “The procedure should also allow for the detection of either lot to lot reagent or calibrator variation, or both, of the examination method.” and “ The use of third-party IQC material should be considered, either as an alternative to, or in addition to, control material supplied by the reagent or instrument manufacturer.”

## What can the reagent manufacturer do to ensure the reliability of calibration?

Under the European Union’s *In-vitro* Diagnostic Directive, the manufacturer is required to demonstrate the traceability of any measurement procedure [7]. This traceability is related to a standard or higher order reference measurement procedure. From this relationship, the calibration results ​​of the manufacturer calibrator are measured. However, the frequency of demonstrating the (continued) traceability property of the calibrator by the manufacturer to higher order reference materials and measurement procedures is not clearly stated in the regulations. Therefore, analytical drift and shift can happen over time despite the initial demonstration of traceability. When such analytical bias occurs, it is generally detected late. In a Norwegian study [8], the glucose concentration in the blood of presumed health subjects and patients with diabetics was measured over a period greater than seven years. In both groups, an increase in glucose concentration was observed. However, this finding was not explained by deterioration in the clinical condition of the subjects, but is related to the lot-to-lot reagent changes that were not corrected by the manufacturer. Recent publications [8, 9] showed that significant differences may occur in reagent lot changes. For this reason, special attention should be given to calibration. Multiple calibrations and the use of independent control material significantly reduce errors and help generate reliable patient data.

## Costs of calibration errors

A study on calibration errors and calcium measurement done in the Mayo Clinic showed the amount of extra costs to be spent if a bias was not corrected [10]. It was determined that calibration error has the potential to lead to bias of 0.1–0.5 mg/dL in up to 15 % of calcium measurements. Furthermore, it was estimated that the cost impact associated with an analytical bias of 0.1 mg/dL could range from 8 to 31 USD per patient (for those receiving a calcium measurement). With approximately 3.55 million patients per year receiving screening serum calcium measurements being affected by a systematic bias, the potential economic impacts range from 60 million to 199 million USD per year for analytic biases of 0.1 and 0.5 mg/dL, respectively.

## Special remarks, which are needed to be taken into consideration

### Commutability, bias, measurement uncertainty, traceability and quality control

Many recently published papers are focusing on measurement uncertainty [11], commutability [9] and on bias vs. reference [12]. Still a clear instruction and advise for doing a proper calibration is missing.

Miller et al. [2]. wrote recently: “An essential attribute for stable metrological traceability is consistency and stability for all elements in the calibration hierarchy, especially when employing a correction for non-commutability. Any changes, e.g., raw materials for ongoing calibrator batches sourced from new vendors, must be carefully assessed before implementation to avoid undetected long-term variations in non-commutability bias.”

We are concentrating in our paper not on quality control procedures [13] as those are done to verify the calibration and therefore are not our focus here.

## Attempt for a recommendation

Currently there are only recommendations on how to proceed calibration provided by the manufacturers of reagents, calibrators and instruments. Therefore, it is essential to adhere strictly to these guidelines as outlined in the instructions for use. However, it’s worth noting that many of these manufacturer recommendations tend to be somewhat minimalistic, aimed at saving time and, consequently, costs. Nevertheless, it is equally important to consider the potential investment of time and resources required for effective troubleshooting in cases where following the manufacturer’s guidance may fall short. In this case an intensive quality control program has to be considered to maintain reliability of test results. There are a reasonable numbers of quality control programs published and in use [13]. The unanswered question here is: Why do designate such extensive resources to quality control procedures while neglecting calibration?

Finally, our recommendation is to do a blanking first and measure at least two calibrators with two different concentrations covering the linear range in duplicates. Non-linear assays may need a different approach. Calibration should be performed whenever modifications are made to the reagent (e.g., using a fresh batch or changing the lot) and/or the instrument (due to maintenance or servicing).

Explanation why: In clinical chemistry assays, a “blank sample” serves as a crucial reference point in the calibration process – so-called blanking. This blank sample replicate all the components found in the sample, except for the specific analyte to be measured. The inclusion of a blank sample is essential to establish a baseline reference and play a pivotal role in eliminating background noise and interference, ensuring that the signal attributed to the analyte remains unaffected by extraneous factors, such as signals from the cuvette (e. g. post washing) or reagents (e. g. color). To maintain measurement accuracy consistently, it is standard practice to include a blank sample in every batch of patient samples analyzed. This practice accounts for potential variations in background noise that may occur over time or across different sets of samples, thereby enhancing the reliability of the assay results.

A two-point calibration with two different concentrations measured in duplicates should be preferred in medical laboratory because it enhances linearity assessment, improves measurement accuracy, detects and corrects errors, increases robustness, and ensures compliance with standards (ISO 15189, CAP, FDA) [6, 14, 15]. The concentrations of the calibrators should cover the analytical linear range of the assay. This approach ultimately leads to more reliable and high-quality results in clinical and research settings.

Remark: Immunodiagnostic assays often exhibit non-linear behavior, requiring a distinct approach. It’s crucial to notice the manufacturer’s instructions for use, and considering duplicate measurements for each calibrator is advisable.

We aim to initiate a dialogue regarding calibration procedures within the medical laboratory, emphasizing the need for increased attention in order to deliver reliable patient results and minimize unnecessary expenses resulting from complex troubleshooting, often stemming from insufficient calibration efforts.

## Definitions as a glossary

For a better understanding, the most important terms are defined below:

### Calibration

Calibration is a process of testing and adjusting an instrument or test system to achieve a correlation between the measurement-response (measurement signal) and the concentration or amount of substance measured by the test procedure. The calibration is a snapshot that does not allow any statements about the temporal behavior (trend, drift) of a measuring system. Only by examining several consecutive calibrations and additional information from quality control measurements can we detect trend and/or drift effects.

### Official calibration

The official calibration includes the quality inspection and marking according to the calibration regulations. Calibrations are prescribed, among other things, for measuring devices for pricing (such as retail scales, fuel dispensers at petrol stations) and for medical devices (e. g. fever thermometer). Since this area is regulated by law, official calibration is carried out by the calibration offices in the individual federal states. The calibrated product receives a verification badge. The process of calibration must be repeated at specified intervals. The term “official calibration” should not be confused with the term “calibration.”

### Adjustment

When adjusting, a measuring system is adjusted or adjusted so that the deviations from the set point are as low as possible and within the device specifications. Adjustment is a process that permanently alters the measuring system. It is often closely related to calibration. The goal of the two processes is to detect deviations, to correct them and to document them. If the display of a measuring system during calibration is outside the permissible tolerances, the unit must be adjusted until the measured values lie within the permissible tolerances.

### Quality control

The quality control serves the continuous monitoring and documentation of the quality of the analytical process, thus also the verification of the calibration. Usually, the control is carried out with the aid of suitable control material. In Germany, control materials with known target values ​​are to be used and the guidelines of Rili-BÄK strictly adhered to. The quality control should ensure that the analysis results are reliable and can be used for both diagnostic and therapeutic questions. It makes a significant contribution to patient safety.

### Measurement

Measuring in our case means determining to what extent a requirement – such as the function or accuracy of a measuring system – is met. The result of the measurement is usually a measured value or a series of measured values. A measurement result is always a more or less precise estimate containing a measurement error. The goal of the measurement is to get an idea about an unknown factor.

### Blank sample

A blank sample states to a reference sample that does not contain the substance or analyte of interest. It is a sample that should ideally have zero concentration or activity of the target analyte.
